# The HAESA family of receptor-like kinases: abscission pathways and emerging functions

**DOI:** 10.3389/fpls.2026.1769235

**Published:** 2026-03-25

**Authors:** Xiang Fan, Rui Geng, Rehman Sarwar, Ke Dong, Jiahui Wen, Xiao-Li Tan

**Affiliations:** 1 School of Life Sciences, Jiangsu University, Zhenjiang, China; 2 School of Food and Biological Engineering, Jiangsu University, Zhenjiang, China

**Keywords:** abscission mechanism, function diversity, HAESA family, IDA (inflorescence deficient in abscission), receptor-like kinases (RLKs)

## Abstract

The HAESA (HAE/HSLs) family of leucine-rich repeat receptor-like kinases (LRR-RLKs) represents a central signaling hub and showed high conservation in multiple plants, integrating developmental cues and environmental stresses. Initially characterized for their pivotal role in floral organ abscission through the IDA-HAE/HSL2 ligand–receptor module, subsequent studies have revealed broader functions in root development, seed longevity, stomatal regulation, and responses to both biotic and abiotic stresses. Structural and genetic analyses have uncovered complex regulatory layers, including membrane trafficking, endoplasmic reticulum output control, co-receptor recruitment, and MAPK cascade activation, all of which ensure precise spatiotemporal control of abscission and related processes. Importantly, the multi-functionality of the HAE family proteins reflects a dynamic trade-off between growth and defense, highlighting its role in balancing resource allocation under complex environments. Overall, the HAESA family not only provides fundamental insights into plant signaling networks but also holds potential as a key target for future crop improvement and sustainable agriculture.

## 1 Introduction

Receptor-like kinases (RLKs) are the largest receptor family in plants and mainly localize at the plasma membrane (Stone et al., 1994; Jose et al., 2020; Soltabayeva et al., 2022). More than 600 RLKs were identified in *Arabidopsis thaliana* and were discovered to participate in regulating multiple physiological processes of plants (Shiu and Bleecker, 2003). RLKs localized at the plasma membrane usually have three domains: the ectodomain (ED) responsible for meditating receptor recognition, the transmembrane domain, and the cytodomain (CD) with kinase activity (Shiu and Bleecker, 2001). When RLKs sensed signals such as hormones or peptides in the apoplast, many RLKs initiate the phosphorylation of intracellular proteins downstream by its cytodomain, followed by MAPK cascade amplification, thereby influencing the related physiological processes effectively (Zhang et al., 2020b). There are around 200 LRR-RLKs (leucine-rich repeat receptor-like kinases) in *Arabidopsis* (Wang et al., 2025), and the name of LRR-RLKs means that there is a leucine-rich repeat within its structure. These LRR-RLKs participated in diverse processes across plant growth, development, and environmental response (Magalhaes et al., 2016; Soltabayeva et al., 2022).

HAESA was initially identified as AtRLK5, a receptor-like kinase characterized in early biochemical studies. *In vitro* kinase assays revealed that AtRLK5 exhibits higher Ser/Thr kinase activity in the presence of Mn^2+^ compared to Mg^2+^; however, whether this preference reflects physiological conditions *in planta* remains unclear (Horn and Walker, 1994; Stone et al., 1994). Several research which followed found that AtRLK5 interacts with different proteins in various species. Then, the function of AtRLK5 was characterized, and its subcellular localization at the plasma membrane and its Ser/Thr protein kinase activity were confirmed. Further analysis revealed that AtRLK5 is highly expressed in mature floral organs while only minimally expressed in the stem and petiole. Owing to the fact that the research into RLKs is gradually underway at that time, in order to accurately distinguish the function of these proteins, the author renamed RLK5 as HAESA. The name “HAESA” is originated from Latin, which means to stick to, adhere to, or cling to, and pointed out that the function of this protein is associated with plant organ abscission (Jinn et al., 2000).

In plants, the detachment of organs is defined as abscission academically (Li and Su, 2024). The abscission process is always linked with cell separation (Merelo et al., 2017; Shi et al., 2019), which is a crucial strategy of energy redistribution (Li and Su, 2024). Plants actively remove old, damaged, or unnecessary organs to prevent the further spread of infection and save energy for other vital physiological processes (Wang et al., 2024)—for instance, plants usually shed some leaves to avoid excessive water loss through reducing transpiration when faced with drought threat. Accordingly, abscission is essential to the completion of plant life cycle (Javed et al., 2017).

Inflorescence deficient in abscission (IDA) encodes a small peptide composed of 77 amino acids (Guo et al., 2021); the *ida* mutant in *Arabidopsis* showed flower shedding defects. The conserved motifs at the C-terminal (EPIP-C) is the common character of IDA and IDLs (IDA-like), and EPIP-C is particularly critical for its function (Butenko et al., 2003; Stenvik et al., 2006). The function of IDA was found to be associated with HAESA and its family member HSL2 (HAESA-Like 2) in the aspect of flower abscission phenotype, but not the same because IDA or its EPIP-C peptide could rescue the abscission defect phenotype of *ida* but cannot rescue *hae hsl2*. Furthermore, the expression patterns of IDA and HAE/HSL2 are highly overlapped, hinting that IDA interacts with HAE/HSL2 while HAE/HSL2 is epistatic to IDA (Cho et al., 2008; Stenvik et al., 2008; Aalen et al., 2013).

Nowadays, research on proteins related to the HAESA family is more extensive, with the main focus still on their role in plant cell separation. Extensive evidence indicates that RLKs mediate diverse plant physiological processes, reflecting the key role of RLKs in plant signal transduction, stress resistance, and other aspects (Zhu et al., 2023). This review article summarized the functions of the HAESA family proteins involved in signaling network and mainly focused on the IDA-HAE/HSL2 module in abscission. In addition, the functional comparison of the HAESA family proteins in different species and their potential research and application prospects were discussed.

## 2 Multi-species phylogenetic analysis of HAESA family proteins

**Figure 1:**
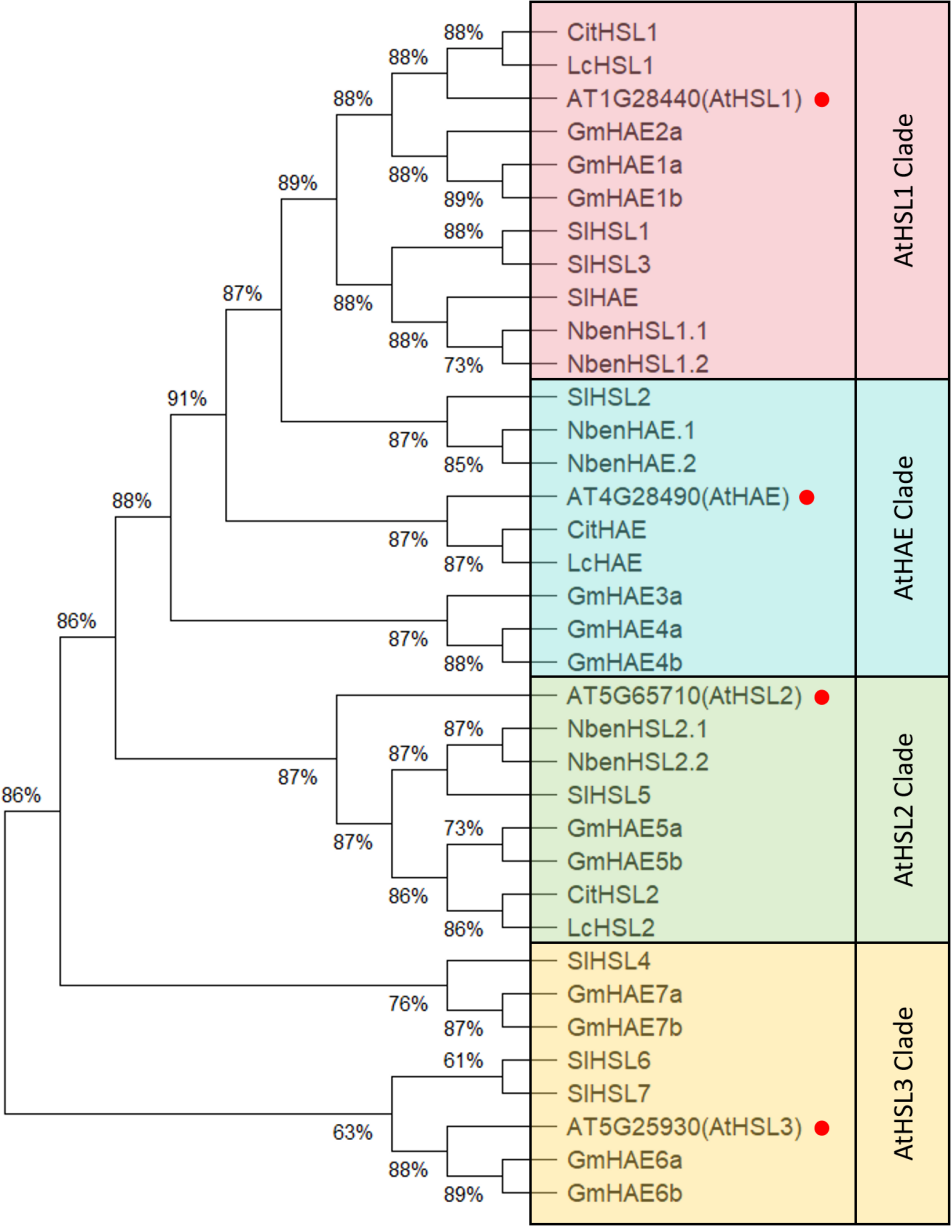
Phylogenetic analysis of HAESA family proteins in different plants. A phylogenetic tree of HAESA family proteins in different plants is constructed by using neighbor-joining (NJ) method with 1,000 bootstrap replicates by MEGA12 (Kumar et al., 2024). All protein sequences were downloaded from Phytozome (Goodstein et al., 2012), NCBI (Sayers et al., 2021), and supplementary data of related literature. The alignment job of protein sequences was done by using ClustalW in MEGA12.

Following numerous reports on HAESA family proteins across plant species in the last 20 years, we selected representative members from updated databases, accounting for revised annotations (**Table 1**). The selected plants comprise *Arabidopsis thaliana*, soybean (*Glycine max*), tomato (*Solanum lycopersicum*), tobacco (*Nicotiana benthamiana*), citrus (*Citrus clementina*), and litchi (*Litchi chinensis*) (Tucker and Yang, 2012; Estornell et al., 2015; Wang et al., 2019a, b; Ventimilla et al., 2020, 2021; Lu et al., 2023; Ma et al., 2024). To investigate the evolutionary relationships within this gene family, we constructed a neighbor-joining (NJ) phylogenetic tree with 1,000 bootstrap replicates (Saitou and Nei, 1987; Zhang and Sun, 2008; Shan et al., 2021). Using the *Arabidopsis thaliana* family members as a reference, homologs from other species were consistently grouped into the corresponding clades, indicating a high degree of conservation across species. Notably, the branching patterns suggest that some clades have species-specific expansion or divergence, providing insights into the potential functional diversification of this gene family during evolution (**Figure 1**).

**Table 1:** List of HAESA family proteins identified in different plants.

Gene name	Gene ID	Typical functions	Reference
*AtHAE*	At4G28490	Floral organ abscission, lateral root emergence, leaf abscission, local immune responses, stomatal movement and drought tolerance	Jinn et al., 2000; Cho et al., 2008; Kumpf et al., 2013; Patharkar and Walker, 2016; Patharkar et al., 2017; Del Corpo et al., 2024; Lalun et al., 2024
*AtHSL2*	At5G65710.1	Floral organ abscission (redundant with *AtHAE*), lateral root emergence, local immune responses, root cap cell separation	Cho et al., 2008; Kumpf et al., 2013; Shi et al., 2018; Lalun et al., 2024
*AtHSL1*	At1G28440.1	CLE9/10 receptor, stomatal development, vascular development, seed germination, and seed longevity	Qian et al., 2018; Li et al., 2019; Chen et al., 2022; Roman et al., 2022
*AtHSL3*	AT5G25930.1	CTNIP/SCREW receptor, drought response, root development, MAPK- and ROS-dependent immune signaling	Qian et al., 2018; Liu et al., 2020, 2022; Rhodes et al., 2022
*NbenHAE.1*	Niben101Scf09774g00001.1	Key receptor sensing NbenIDA1 in regulating corolla abscission	Ventimilla et al., 2020, 2021
*NbenHAE.2*	Niben101Scf05190g00001.1	Non-AZ specific expression, function uncertain	
*NbenHSL1.1*	Niben101Scf03169g02005.1		
*NbenHSL1.2*	Niben101Scf11552g02006.1		
*NbenHSL2.1*	Niben101Scf08143g03001.1		
*NbenHSL2.2*	Niben101Scf02417g01010.1		
*CitHAE*	Ciclev10014127m	Floral organ and fruit abscission	Estornell et al., 2015; Magalhaes et al., 2016
*CitHSL1*	Ciclev10014813m		
*CitHSL2*	Ciclev10026946m		
*GmHAE1a*	Glyma.13G174900.1	Non-AZ specific expression, function uncertain	Tucker and Yang, 2012
*GmHAE1b*	Glyma.07G201600.4		
*GmHAE2a*	Glyma.13G235000.1	Likely induced in ethylene-mediated leaf senescence	
*GmHAE3a*	Glyma.06G288600.1	Non-AZ specific expression, function uncertain	
*GmHAE4a*	Glyma.13G294100.1		
*GmHAE4b*	Glyma.12G206800.1		
*GmHAE5a*	Glyma.01G197600.1		
*GmHAE5b*	Glyma.11G040200.1		
*GmHAE6a*	Glyma.04G086700.1		
*GmHAE6b*	Glyma.06G088400.1		
*GmHAE7a*	Glyma.06G090800.1		
*GmHAE7b*	Glyma.04G088800.1		
*SlHAE*	Solyc02g077630.2.1	Non-AZ specific expression, pathogen-induced defense response	Tucker and Yang, 2012; Wang et al., 2017; Lu et al., 2023
*SlHSL1*	Solyc03g006300.1.1		
*SlHSL2*	Solyc07g053600.2.1		
*SlHSL3*	Solyc03g006300.1.1	Non-AZ specific expression, function uncertain	
*SlHSL4*	Solyc04g077010.2.1		
*SlHSL5*	Solyc02g091860.2.1		
*SlHSL6*	Solyc08g066270.1.1	AZ specific expression, likely induced in abscission	
*SlHSL7*	Solyc08g066320.2.1		
*LcHAE*	LITCHI029130.m1	Likely involved in developmental regulation rather than abscission	Wang et al., 2019a; Ma et al., 2024
*LcHSL1*	LITCHI029130.m1		
*LcHSL2*	LITCHI007137.m1	Floral organ and fruitlet abscission, auxin/ethylene response	

## 3 Mechanism of HAESA family proteins in abscission

Constructions of anti-sense HAESA transgenic lines suggest that the transcription of HAESA is positively associated with flower shedding rate, hinting that this protein serves as a key component in plant flower abscission regulation (Jinn et al., 2000; Du et al., 2021). When *ida* mutants from *Arabidopsis thaliana* were screened and the gene *IDA* was identified, it was discovered that the flower organ abscission defect caused by IDA is not affected by ethylene (ET) and is controlled by a single recessive gene (Butenko et al., 2003, 2006). The initial discovery of IDA identified its role as a signaling peptide, although its corresponding receptor remained elusive. Subsequent analyses suggested that IDA was most likely recognized by an LRR-RLK. This led to the identification of its two receptors with highly similar tissue-specific expression patterns: HAE (HAESA) and HSL2 (HAESA-LIKE 2) (Cho et al., 2008; Stenvik et al., 2008). Functional validation demonstrated that the *hae hsl2* double mutant displayed the phenotype of non-shedding flowers, characterized by the persistent attachment of petals, sepals, and stamens to the plant (Lee et al., 2022). In contrast, the *hae* or *hsl2* single mutant line exhibited no obvious phenotype. Notably, IDA was unable to rescue the phenotype of non-shedding flowers in *hae hsl2* (Zhang and Zhang, 2022), thereby providing compelling evidence that IDA peptides and HAESA family receptors constitute the central ligand–receptor module governing cell separation at abscission zones in flower shedding (Stenvik et al., 2008; Aalen et al., 2013).

**Figure 2:**
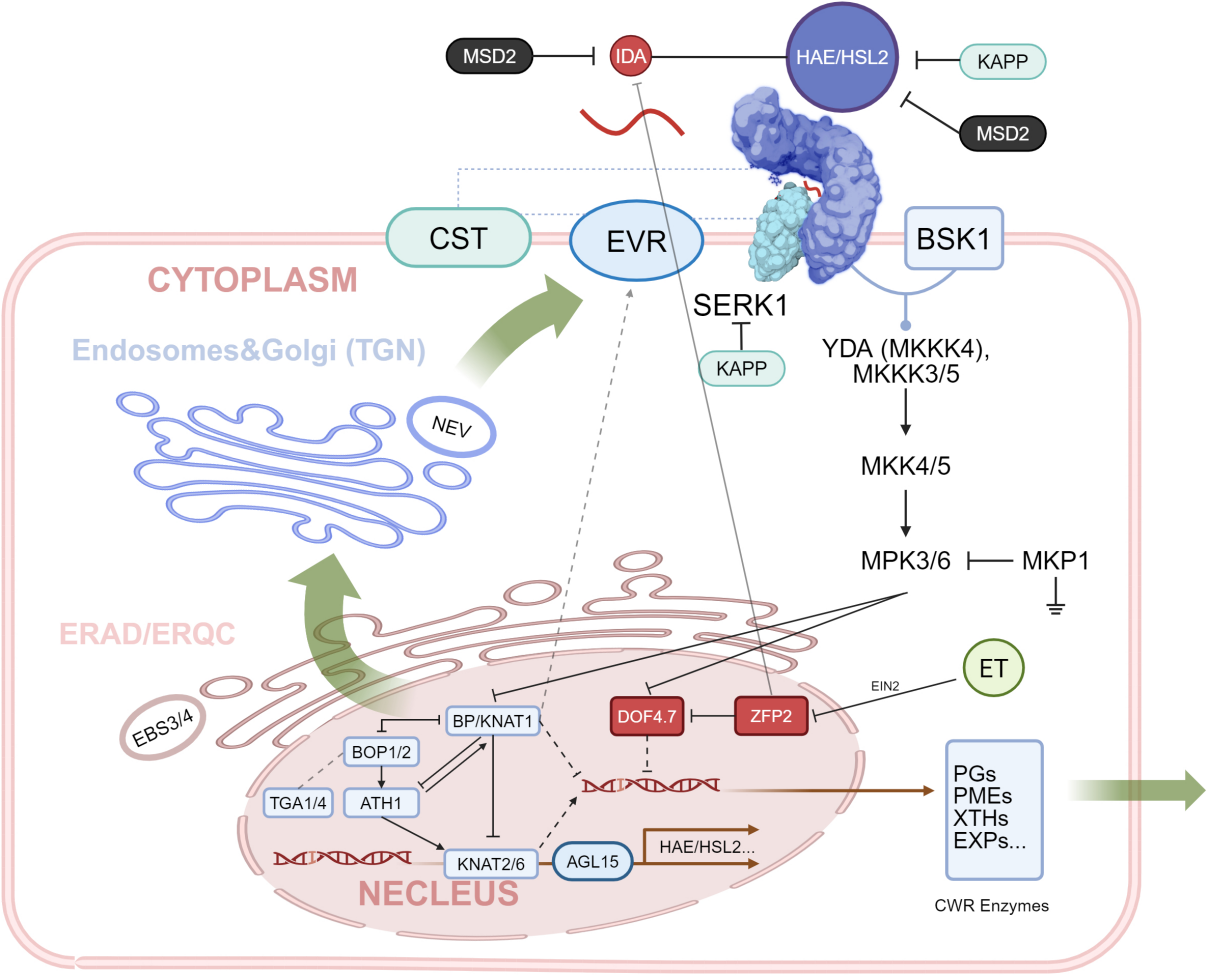
Regulatory network of abscission centered on IDA-HAE/HSL2 module. IDA is sensed by receptor HAE/HSL2 under the assistance of co-receptors centered by SERK1, thereby transmitting the abscission signal into the plasma membrane. The abscission signal is transmitted to the MAPK cascade (YDA-MKK4/5-MPK3/6) with the help of scaffold protein BSK1. Amplified signal is transmitted into the cell nucleus and regulates a series of transcription factors (TFs) such as DOF4.7, BOP1/2, KNAT1, and AGL15, thereby influencing the expression of HAE/HSL2 and CWR genes. Primarily folded HAE/HSL2 receptors require ERAD/ERQC to ensure correct output. Vesicle transportation with participants located both on the Golgi (NEV) and plasma membrane (EVR, CST) regulates the abundance of receptors on the plasma membrane, thereby regulating the efficiency of abscission signal transmission.

Crystallographic analyses further revealed that the IDA peptide specifically occupies the ligand-binding pocket of the HAE receptor, forming a stable ligand–receptor complex that subsequently activates downstream signaling cascades (Santiago et al., 2016). Collectively, these findings established the IDA-HAE/HSL2 module as a central hub for cell separation and organ detachment in plants (Liu et al., 2013). With the accumulation of related researches, an increasingly comprehensive signaling network has emerged, centered on this ligand–receptor pair and shaping our recognition of the molecular framework governing abscission (**Figure 2**).

### 3.1 Abscission efficiency regulated by membrane vesicle transportation

The correct localization of membrane proteins requires processing and sorting through the ER (endoplasmic reticulum) and Golgi apparatus, followed by vesicular transportation to the plasma membrane. In addition, regulation of plasma membrane composition involves endocytosis, which internalizes membrane proteins and lipids via endosome formation for subsequent recycling or degradation (Gomez-Navarro and Miller, 2016). These endosomes can then be degraded to generate metabolites needed by the cell while simultaneously ensuring the balance and homeostasis of receptor abundance and membrane components both at the cell surface and within the intracellular environment (O’Sullivan and Lindsay, 2020).

*NEVERSHED* (*NEV*) encodes an ATP–ribosylation factor–GTP-activating protein (Liljegren et al., 2009; Liu et al., 2013) and is primarily localized to the Golgi apparatus and the ER. *nev* exhibited pronounced defects in floral organ abscission. At the subcellular level, these mutants showed a disrupted Golgi structure, and vesicles accumulated between the plasma membrane and the cell wall (Leslie et al., 2010). Taken together, these findings suggested that NEV affects abscission by disrupting intracellular trafficking (Liljegren et al., 2009).

Subsequently, the *EVERSHED* (*EVR*) gene which encodes an LRR-RLK was identified through EMS mutagenesis screening (Kondo et al., 2014). *evr* mutants were able to rescue the Golgi and trans-Golgi network (TGN) defects caused by *nev*, ultimately restoring the abscission defect, indicating that EVR is playing an inhibitory role in abscission (Zhao et al., 2021). In *nev evr* double mutants, abscission zone (AZ) cells expanded and the AZs were enlarged, but abscission still proceeded normally. Because EVR is localized at the plasma membrane and exhibits a similar transcription pattern in AZs to HAE and HSL2, EVR was hypothesized to interact with HAE/HSL2 and influences abscission by modulating their membrane trafficking (Liljegren et al., 2009; Leslie et al., 2010; Liu et al., 2013).

Importantly, in *hae hsl2*, the phenotype of *nev* and *evr* were not observed (Gou and Li, 2020), indicating that their functions depend on HAE/HSL2 activity and are independent from IDA (Gubert and Liljegren, 2014). Collectively, these findings support a model in which NEV promotes abscission either by directly meditating the delivery or retrieving of HAE/HSL2 from the plasma membrane or by indirectly promoting the removal of EVR, thereby allowing the effective transmission of the abscission signal (Liu et al., 2013; Gubert and Liljegren, 2014).

Two kinase-encoding genes, *SERK1* (*Somatic Embryogenesis Receptor Kinase 1*) and *CST* (*CAST AWAY*) were further identified (Burr et al., 2011; Groner et al., 2016). Mutations in either gene were able to rescue the non-shedding flower phenotype in *nev*, although the enlarged abscission zone with cell expansion phenotypes persisted (Wang et al., 2019a). Notably, *serk1* and *cst* mutations could not rescue the abscission defects in *ida* or *hae hsl2*, indicating that SERK1 and CST regulation may parallel with the IDA-HAE/HSL2 signaling pathway (Liljegren et al., 2009). Consistent with this hypothesis, BiFC assays demonstrated that CST straightly interacts with HAE and EVR at the plasma membrane, while the function of SERK1 was confirmed and will be illustrated in detail later (Lewis et al., 2010; Burr et al., 2011; Gubert and Liljegren, 2014). CST shows its localization predominantly on plasma membrane, while EVR exhibits both at the plasma membrane and intracellular structures and the CST–EVR complex appears in the same position at the cell surface (Liljegren et al., 2009). It was suspected that CST may act as an anchor of EVR at the plasma membrane. Because SERK1, CST, and EVR all localize to the plasma membrane, it is further hypothesized that beyond recognition of IDA, CST and EVR may dissociate from the core receptors HAE and HSL2, thereby permitting downstream abscission signaling to proceed (Liljegren et al., 2009; Niederhuth et al., 2013a; Groner et al., 2016; Patharkar and Walker, 2018).

In certain weak *hae hsl2* mutants, protein misfolding may occur while retaining part of its function. However, plant cells possess strict quality-control systems to prevent the export of misfolded proteins. Within ER, for example, the ER-associated degradation (ERAD) and ER quality control (ERQC) could support accurate protein folding while enabling the retrieval of improperly folded proteins (Gomez-Navarro and Miller, 2016; Strasser, 2018; Zhao et al., 2023b). *EBS3/4* (*EMS-MUTAGENIZED BRI1 SUPPRESSOR 3* and *4*) genes, which both encode mannosyltransferases, are involved in the ERAD pathway (Baer et al., 2016). In the weak *hae hsl2* background, mutation of either gene could rescue flower abscission. In WT, properly folded proteins are exported after recognition, whereas misfolded proteins are delivered to degradation (Sun and Brodsky, 2019). In *ebs3* and *ebs4*, incomplete N-glycan assembly prevents the exposure of the α1,6-linked mannose residue required for recognition. As a result, some misfolded but still partially functional HAE/HSL2 receptors escape degradation, accumulate at the plasma membrane, and thereby rescue the abscission defect of *hae hsl2*. This module described how ERAD and ERQC influence abscission by regulating the folding and sorting of HAE and HSL2 receptors (Hong et al., 2012; Baer et al., 2016; Zhang et al., 2020a).

In summary, this group of regulators influences flower abscission by altering membrane trafficking and, in particular, by affecting the accumulation of functional HAE and HSL2 receptors, thereby altering the efficiency of the IDA-HAE/HSL2 signaling module (Wang et al., 2019a; Cui et al., 2022). Initially, the receptor proteins HAE and HSL2 undergo folding and processing in the ER, where correctly folded receptors, as well as partially functional proteins “released” by EBS3 and EBS4, are exported. In the Golgi, NEV plays a central role in vesicular transport in TGN, ensuring the delivery of HAE and related receptors to the plasma membrane. At the cell surface, EVR with some “anchors” possibly mediate the endocytosis of these receptors, reducing their abundance at the membrane and repressing the activity of NEV. This balance explains why *nev evr* double mutants restore Golgi function. In *nev evr*, functional receptors successfully reach the plasma membrane but cannot be recycled back into the cell due to the loss of EVR function, resulting in excessive accumulation at the cell surface and, consequently, premature floral organ abscission.

### 3.2 Participants in abscission in downstream MAPK cascade

Within downstream of IDA-HAE/HSL2 signaling, the MAPK cascade serves as a key signal amplification axis, involving different hierarchies of protein kinases typically organized into three tiers: MKKKs (MAPK kinase kinases), MKKs (MAPK kinases), and MPKs (MAP kinases) (Jagodzik et al., 2018; Patharkar and Walker, 2018; Chen et al., 2024).

Activation of HAE/HSL2 triggers the phosphorylation and activation of MKKK family member YDA, which, in turn, activates MKK4/5. These MKKs subsequently phosphorylate and activate the downstream MPK3/6, the terminal effectors of the cascade. MPK3 and MPK6 influence diverse gene expression through phosphorylation of downstream substrates, including various cell wall remodeling (CWR) enzymes—for instance: PGs, PMEs, XTHs, EXPs, etc. The induction of CWR enzymes facilitates cell wall degradation, thereby promoting cell separation triggered by abscission zone (AZ) cells (Cho et al., 2008; Lashbrook and Cai, 2008; Patharkar and Walker, 2018; Qiu et al., 2021).

YDA (MKKK4) has previously been shown to participate in the control of embryogenesis, stomatal development, and immune responses (Tellez et al., 2020; Wu et al., 2024). Mutants such as ydaTSKO exhibit defects in abscission, while a constitutively active version of YDA (YDAca+SSP, where SSP, a BSK protein, can activate endogenous YDA independently of membrane receptors in SSP-expressing cells) was shown to rescue both the abscission defects and abnormal AZ phenotypes of *ida* and *hae hsl2* (Galindo-Trigo et al., 2024). These findings establish YDA as a critical downstream regulator of IDA and HAE/HSL2 signaling. In addition, double mutants *mapkkk3 mapkkk5* displayed various degrees of abscission defects that are similar to ydaTSKO, indicating that although functional redundancy exists among these MKKKs, YDA exerts a predominant role in this pathway. In addition, the YDA-MKK4/5-MPK3/6 axis could also be activated by RGF1-RGI ligand–receptor pair, indicating the function redundancy of this MAPK cascade (Shao et al., 2020; Wang et al., 2022; Galindo-Trigo et al., 2024).

In addition to the core kinase cascade, BSK1 functions as a positive regulatory scaffold that facilitates signal transmission from HAE/HSL2 to YDA. BSK1 (Brassinosteroid Signaling Kinase 1), a RLK which localized at the plasma membrane, is involved in multiple physiological processes and interacts directly with HAE and HSL2 receptors (Liljegren et al., 2009; Galindo-Trigo et al., 2024). Notably, during abscission, BSK1 does not act through its kinase activity; it functions instead as a scaffold protein which mediates the assembly of the HAE-BSK1-YDA signaling module, thereby resulting in the engagement of downstream MAPK cascade while ensuring robust signal amplification (Sun and Zhang, 2022; Galindo-Trigo et al., 2024; Li and Su, 2024).

To ensure that the abscission signal is not misinterpreted and is terminated in a timely manner, plants employ mechanisms similar to grounding wires and breakers in electrical circuits, whereby MKP1 restrains basal MAPK activity and KAPP dephosphorylates activated receptor complexes. These mechanisms prevent the initiation of abscission when the signal remains below the noise threshold and shut down the IDA signal immediately once the organ has separated from the plant. MKP1 functions as a negative regulator of abscission signaling by restricting basal MAPK activity prior to pathway activation, thereby establishing a signaling threshold that prevents inappropriate or premature abscission (Taylor et al., 2024). At the same time, IDA-family peptides (IDL1/2/3) are expressed only on the secession side of the AZ in flowers, while HAE/HSL2 and MKP1 are expressed on both the secession sides and residuum sides. Once the floral organs detach, ligand production ceases, thereby preventing the prolonged activation of abscission signal (Cho et al., 2008; Bartels et al., 2010; Meng et al., 2016; Taylor et al., 2024). Interestingly, in early research, KAPP (kinase-associated protein phosphatase), which is a PP2C that negatively regulates receptor-like kinase signaling, has a similar function (Becraft, 2002; Shah et al., 2002; Manabe et al., 2008; Lu et al., 2020). In IDA-HAE/HSL2, KAPP interacts with the phosphorylated cytoplasmic domains of HAE and SERK1, dephosphorylating them to terminate signal transmission. This mechanism prevents the sustained activation of the MAPK cascade and ensures in-time control of organ abscission. KAPP and SERK1 co-localize at the plasma membrane and early endosomes, suggesting the function in receptor internalization and recycling (Shiu and Bleecker, 2001; Shi et al., 2011; Taylor et al., 2016; Wang et al., 2023). These multilayered regulatory mechanisms ensure that the MAPK cascade is precisely initiated and amplified during abscission while avoiding inappropriate or overactive signaling.

After the end of the MAPK cascade, transcription factors ZFP2 (C2H2 zinc finger transcription factor) and DOF4.7 (DNA-binding with one finger) form an antagonistic regulatory pair (Kim et al., 2015; Wang et al., 2016). DOF4.7 functions as a repressor of abscission by downregulating the expression of downstream CWR enzymes, which could be degraded and destabilized by MPK6 (Crick et al., 2022; Wang et al., 2023). ZFP2 acts upstream to inhibit DOF4.7 transcription, further enhancing CWR enzyme expression and organ separation by introducing ET, which link the ET pathway and IDA-HAE/HSL2 module together and will be further explained later (Wei et al., 2010; Shi et al., 2019; Wang et al., 2023).

### 3.3 Transcription factors involved in IDA-HAE/HSL2 module

The expression of HAESA is tightly regulated in both temporal and spatial dimensions, showing a high expression only during specific stages of floral organ abscission. This strongly suggests the presence of additional components and regulatory mechanisms that influence its activity such as transcription factors (TFs).

The expression of HAESA increases by approximately 27-fold between floral stages 12 and 15, suggesting the existence of a positive feedback loop within the signaling pathway. Promoter analysis of 100 co-expressed genes revealed the enrichment of cis-elements bound by *AGL15* (*AGAMOUS-like 15*) and members of the *WRKY* family (W-box), indicating that these transcription factors are likely to regulate HAESA expression. AGL15, a MADS-box transcription factor, is widely participated in hormone signaling and plays essential roles during plant development. In *MKK4/5* RNAi lines, the phosphorylation of a specific AGL15 isoform was reduced, accompanied by a decreased HAESA transcript level, indicating that MAPK signaling is required to relieve the AGL15-mediated repression of HAESA transcription. *In vitro* assays further demonstrated that over-expression of AGL15 delays floral organ abscission, which is consistent with its role as a transcriptional repressor. Overall, these observations lead to the following conceptual framework: upon activation of the abscission signal, the IDA-HAE/HSL2 module stimulates the MAPK cascade, in which MKK4/5-mediated phosphorylation suppresses the repressive activity of AGL15. This relieves the inhibition of HAESA transcription and thereby amplifies HAESA expression, forming a positive feedback loop which guarantees a robust activation of the abscission signal precisely when required (Patharkar and Walker, 2015; Patharkar et al., 2017).

An abnormal expression of IDA results in premature cell separation and abscission, indicating the necessity of negative feedback regulators to spatially and temporally control the activation of the abscission signal (Taylor et al., 2024). Mutations of *BP/KNAT1* rescued the abscission defects of *ida* floral organs. Other members of the same family, KNAT2 and KNAT6, were found to exhibit expression patterns similar to BP/KNAT1. In *bp/knat1*, the abscission zone (AZ) was enlarged, and the electron microscopy results resembled those observed in IDA-overexpression lines. In contrast, *knat2/6* displayed AZ morphology comparable to the WT. An analysis of multiple mutants suggested that BP/KNAT1 functions downstream of IDA-HAE/HSL2, while KNAT2 and KNAT6 play antagonistic roles against BP/KNAT1 in regulating floral organ abscission (Shi et al., 2011; Butenko et al., 2012). The KNAT family proteins typically act as transcription factors in that the authors suggest BP/KNAT1 and KNAT2/6 function downstream of the MAPK cascade within the IDA-HAE/HSL2 module. In this model, BP/KNAT1 serves as a repressor, likely by holding the activity of the positive regulators KNAT2 and KNAT6. Interestingly, *bp/knat1* mutants also show expanded abscission zones. Although BP/KNAT1 has been reported to interact with EVR, its potential involvement in HAE/HSL2 trafficking remains speculative. It is therefore possible that BP/KNAT1 influences abscission by modulating the spatial organization or signaling context of the abscission zone, which may indirectly affect the output of the IDA-HAE/HSL2 pathway (Shi et al., 2011; Butenko et al., 2012). Further investigation will be required to determine whether BP/KNAT1 has a direct role in regulating HAE/HSL2 trafficking or acts primarily through transcriptional and spatial control mechanisms.

Transcription factors BOP1/2 (BLADE-ON-PETIOLE 1 and 2) belong to BTB/POZ domain family (Hepworth et al., 2005). *bop1 bop2* have flower abscission defect, and AZ cells do not differentiate, which cannot be rescued by IDA, indicating that BOP1/2 function upstream of the IDA-HAE/HSL2 module (Ma et al., 2023). Functionally, BOP1/2 repress BP/KNAT1, activate KNAT2/6 by upregulating *ATH1* (*ARABIDOPSIS THALIANA HOMEOBOX GENE 1*), and indirectly promote the expression of CWR enzymes, which execute cell separation during abscission (McKim et al., 2008; Crick et al., 2022). These genes collectively mediate cell separation during organ shedding.

### 3.4 Co-receptor kinases SERKs and functions related to HAESA family

The Somatic Embryogenesis Receptor Kinase (SERK) family in *Arabidopsis* is composed of five known members (SERK1-SERK5), many of which participate in diverse developmental processes including embryogenesis, cell division, and immune responses. SERKs generally do not act as independent receptors but function as co-receptors, forming complexes with other receptor kinases such as RLKs to enhance signaling efficiency (Wu et al., 2015; Li et al., 2019). SERK1 serves as a shared co-receptor that engages in different ligand–receptor interactions, such as BRI1 for brassinosteroid perception and HAESA for IDA-mediated abscission. Within the IDA-HAE/HSL2 module, SERK1 is recruited after the initial low-affinity between IDA and HAESA (Kd ≈ 20 μM) and then forms a relatively stable complex with high affinity (Kd ≈ 0.3 μM). This ligand-induced association promotes kinase transphosphorylation and activates MAPK signaling to trigger floral organ abscission (Santiago et al., 2016; Hohmann et al., 2017, 2018).

Multiple mutants of the SERK family (*serk1/2/3* or *serk1/3/4*) exhibited abscission defects which are similar to the phenotype of *hae hsl2* mutants. Consistent with this phenotype, SERKs were reported to be expressed in AZs, hinting their potential functional redundancy (Meng et al., 2016). In the same year, the crystal structure of the IDA-HAESA-SERK1 complex was revealed (Okuda, 2021), which further confirms that SERK1 functions as a co-receptor by enhancing the binding affinity between IDA and HAE. Subsequent structural and molecular biological studies further demonstrated that members of the HAESA receptor family always depend on SERK co-receptors for the recognition of IDA-family ligands (IDA and IDLs) (Meng et al., 2016; Santiago et al., 2016; Gou and Li, 2020).

## 4 Role of HAESA family in plant physiological processes

**Figure 3:**
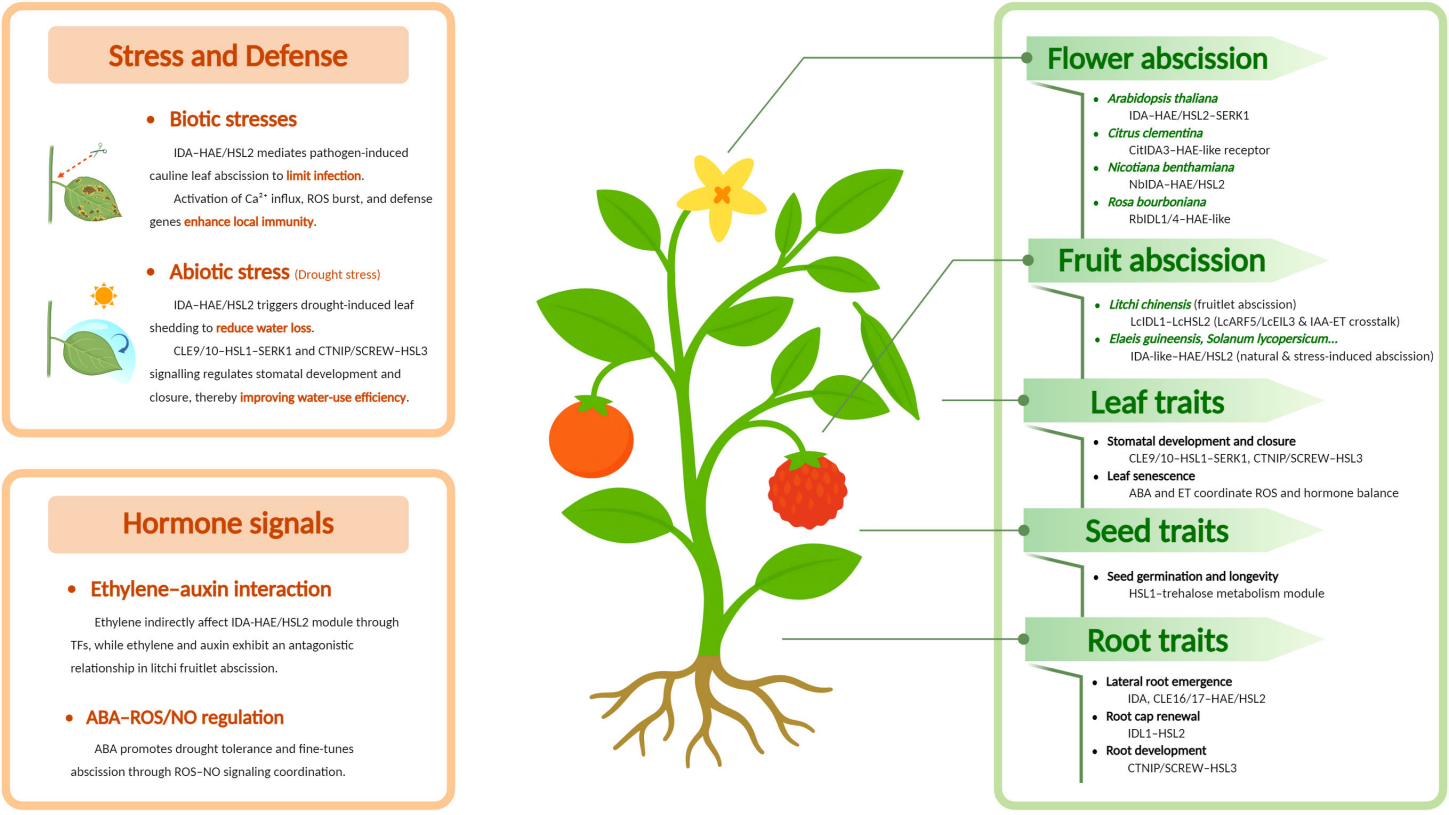
Functional landscape of HAESA family receptor kinases. Overview of HAESA family-mediated regulation of abscission, stress responses, hormone signaling, and developmental traits in different plant organs.

Despite the core function in regulating the abscission process in plants, HAESA family proteins also participate in various aspects, which means that they not only serve as the controller of abscission but their function extends from single-cell layer separation to organ-level defense and is deeply engaged in overall plant development and environmental adaptation (**Figure 3**).

### 4.1 Defense response to biotic and abiotic stresses

#### 4.1.1 The dual function of HAESA family proteins in response to biotic stresses

When *Arabidopsis* leaves are infected by *Pseudomonas syringae*, HAE/HSL2 mediate the abscission of the cauline leaves, while in *hae hsl2* pathogen-induced cauline leaf abscission is nearly abolished, indicating that HAESA possibly contributes to plant immunity by shedding infected leaves to prevent the further spread of the pathogen (Patharkar et al., 2017; Wang et al., 2017). Recognized by HAE and HSL2, IDA can trigger Ca^2+^ influx and ROS production, which are symbols of early immune responses. Besides that, the activation of multiple defense response genes such as *FRK1*, *MYB51*, and *PEP3* also represents the arousal of a defense reaction (Pautot et al., 2025). Moreover, the expression of IDA itself is induced by both biotic and abiotic stresses (Lalun et al., 2024), highlighting that IDA-HAE/HSL2 is not only involved in abscission but also may activate local immune responses, thereby providing a dual layer of protection (Olsson et al., 2019; Del Corpo et al., 2024; Lalun et al., 2024).

Pathogens could exploit HAESA-related functions to facilitate their infection—for example, Hs2D01, a cytoplasmic effector, interacts with the cytodomain of HAE, potentially interfering with HAE-mediated CWR gene expression and promoting the formation of nematode feeding sites (syncytia) (Verma et al., 2022). In tomato, Pst DC3000 may indirectly activate the HAE/HSL2-ADPG2 module by upregulating IDL6, leading to enhanced pectin degradation in the cell wall which could reduce pathogen resistance and make it easier for infection (Wang et al., 2017).

#### 4.1.2 HAESA family proteins regulate plant response to abiotic stress

In addition to their established roles in developmental regulation, HAESA family receptors have increasingly been implicated in plant responses to abiotic stress (Ou et al., 2021; Gandhi and Oelmuller, 2023). These responses often involve the coordinated control of stomatal behavior, hormone signaling, and organ abscission to optimize water use and stress tolerance (Kim et al., 2021; Cui et al., 2023; Matkowski and Daszkowska-Golec, 2023; Shahani et al., 2023). Recent studies suggest that HAE/HSL-mediated peptide signaling intersects with ABA and MAPK pathways, enabling plants to balance both physiological and developmental processes under diverse environmental conditions.

CLE9/10 (CLAVATA3/ESR-RELATED 9 and 10) peptides are recognized by HSL1 (with binding affinities of Kd ≈ 40 μM directly and Kd ≈ 1.5 μM in the presence of co-receptor SERK1). This signaling module downregulates the formation of stomatal precursor cells (MMCs) by promoting MAPK-mediated phosphorylation of the main stomatal transcription factor SPCH (SPEECHLESS), thereby accelerating its degradation and inhibiting MMC formation. Among the two peptides, CLE9 plays the predominant role (Qian et al., 2018).

Drought stress induces cauline leaf abscission in *Arabidopsis* through the IDA-HAE/HSL2 module (Man et al., 2023), reducing transpirational surface area and enhancing drought tolerance. Upon rewatering, the same pathway triggers leaf shedding, highlighting its role in drought resistance (Patharkar and Walker, 2016). Moreover, HSL3 has also been involved in drought resistance through the SCREW-NUT (CTNIP-HSL3) signaling module. In this module, activation of the SCREW-NUT complex triggers the NUT-dependent phosphorylation of the PP2C-type phosphatases ABI1/ABI2 (ABA INSENSITIVE 1 and 2), which inhibit OST1 (OPEN STOMATA 1) activity, suppressing ABA-induced activation of S-type anion channels such as SLAC1 (Slow Anion Channel-Associated 1) and consequently preventing stomatal closure. However, both SCREW (CTNIP4) and NUT (HSL3) are induced by MAMPs, ABA, drought, and pathogen infection, which makes the corresponding mutants more sensitive to pathogens (Liu et al., 2022; Rhodes et al., 2022).

This signaling route is also closely related to H_2_O_2_ homeostasis. HSL3 negatively regulates ABA signaling by suppressing RbohF activity and reducing CAT activity, thereby altering H_2_O_2_ balance. Notably, this function is possibly overlapped with that of HSL1-CLE9, hinting the existence of functional redundancy among HAE family receptors. Collectively, these findings indicate that the interplay between HSL1/HSL3 and plant hormones is related to drought resistance (Qian et al., 2018; Liu et al., 2020).

### 4.2 Regulation of root-related traits

HAE/HSL2 serves a vital function in lateral root emergence (LRE) (Kumpf et al., 2013; Zhu et al., 2019). During this process, lateral root primordia (LRP) must penetrate three overlying tissue layers—the endodermis (EN), the cortex (CO), and the epidermis (EP)—requiring precise cell wall degradation without disturbing the integrity of the primary root (Zhu et al., 2019). At the early stages of LRE, auxin-induced upregulation of IDA activates IDA-HAE/HSL2, which, in turn, regulates CWR genes (e.g., PGs, EXPs) through the MAPK cascade. This promotes pectin degradation and loosening of the cell walls covering the LRP, thereby facilitating their penetration through the cover. In *hae hsl2* mutants, these cell layers remain intact and significantly reduce lateral root density, underscoring the important role of HAE/HSL2-mediated signaling in this process (Kumpf et al., 2013; Lalun et al., 2024).

IDL1 is recognized by HSL2 and may induce CWR and PCD (programmed cell death) in the outer cell layer through the MAPK cascade, finally leading to its overall shedding (Zhao et al., 2021; Sun and Zhang, 2022). At the same time, this pathway promotes the differentiation of inner cells, thereby controlling the renewal of the cell layer on the top of root caps. As a central regulatory module, the IDL1-HSL2 module controls the balance between root cap cell differentiation and abscission, ensuring that the root cap is maintained at approximately six cell layers in thickness (Shi et al., 2018; Zhu et al., 2019).

Peptides CLE16/17 promote LRE by upregulating the expression of HAE/HSL2, thereby accelerating the transformation of LRP from early-stage primordia to epidermal penetration. In addition, CLE16/17 rely on the CLV1-ACR4 complex to promote stem cell differentiation (Zhao et al., 2021). These findings indicate that CLE16/17 can also regulate both root cap renewal and lateral root density simultaneously and independently (Zhang et al., 2022).

Peptides of the CTNIP (C-terminally nitrated peptide) family function as signaling peptides that are recognized by HSL3 with the assistance of the co-receptor SERK3 (BAK1). Activation of this pathway inhibits root growth, leading to root skewing, while SERK involvement in the process further contributes to root curling (Rhodes et al., 2022). Another study also reported the SCREW (Small Cytokines Regulating Water Loss) peptide, pointing out that it could be responded by HSL3 and functions in drought response and plant immunity by regulating stomatal closure (Liu et al., 2022).

### 4.3 Regulation of seed-related traits

The mutant *Athsl1* line presents a lower germination rate, while the AtHSL1 over-expression line presents a higher germination rate, and the RNA-seq data are indicating that HSL1 positively regulates *Arabidopsis* seed lifespan by regulating trehalose synthesis, pectin metabolism, and the antioxidant system. This study expanded the range of function of HAESA family proteins and mentioned the relationship between HAESA family proteins with seed longevity for the first time (Chen et al., 2022). *hae hsl2* and *ida* lines did not significantly decrease the seed productivity, indicating that HAE/IDA is a potential agricultural improvement target that can regulate abscission without affecting yield (Patharkar and Walker, 2016).

### 4.4 HAESA family proteins in plant hormone networks

Changes in plant hormone levels engage in diverse physiological processes in plants and are also an indirect reflection of the physiological state of plants (Verma et al., 2016; Zhao et al., 2021; Zhang et al., 2023). HAE family proteins are not only passive receptors that identify ligands; they also contribute significantly in the plant hormone network.

#### 4.4.1 HAE/HSL2

HAE/HSL2 are the key characters in the abscission pathway and the main response elements of plant hormones. ET (ethylene) is a plant endogenous gaseous hormone with simple structure and high activity, regulating various procedures including plant development and resistance (Dubois et al., 2018). The abscission pathway is reported in parallel with the ET pathway firstly in 2003 (Butenko et al., 2003) and was further corrected in later research because we found that ET indirectly affects the IDA-HAE/HSL2 module. Furthermore, we discovered that ET might be involved in the modulation of CWR enzyme expression and contribute to the abscission process.

Recent findings of transcription factor ZFP2 and DOF4.7 exhibit a crosstalk between IDA-HAE/HSL2 module and ET, which was contrary to the previous suspicion that these pathways act independently (Kim et al., 2015; Crick et al., 2022). Participants in the ethylene pathway such as EIN2 temporally repress ZFP2, thereby activating DOF4.7, suppressing CWR enzymes such as ADPG2, and preventing premature cell separation (Wang et al., 2016; Crick et al., 2022). Within the IDA-HAE/HSL2 module, DOF4.7 could be phosphorylated and be degraded by MPK6, while ET could induce DOF4.7 expression (Wang et al., 2016; Xu et al., 2020; Crick et al., 2022). A similar regulation model is confirmed in rose (*Rosa chinensis* Jacq. cv. ‘Gold Medal’) (Gao et al., 2016). This dual-regulation ensures the precise timing of flower detachment by bridging ET signaling and the IDA-HAE/HSL2 pathway.

During LRE, the auxin (IAA) levels are high at first but decline when the LRP approaches the epidermis. This reduction in IAA coincides with IDA expression, which activates the IDA-HAE/HSL2–MAPK axis to promote CWR enzyme expression and facilitate LRP penetration through the outer cell layers (Kumpf et al., 2013).

Recent studies have revealed that ROS-related regulators also participate in regulating the timing of abscission initiation. Among them, MSD2 (MANGANESE SUPEROXIDE DISMUTASE 2), a secreted Mn-superoxide dismutase preferentially expressed in the floral abscission zone, acts as an upstream modulator of the IDA-HAE/HSL2 pathway. When the abscission process is about to start, the absence of MSD2 results in the earlier position of floral organ abscission by influencing ABA and NO response, in which ONOO^-^ could upregulate the ABA response gene and *vice versa*, forming a positive feedback loop. These upstream elements could guarantee that the abscission process is being launched at the appropriate time (Lee et al., 2022).

RNA-Seq of stage-15 receptacles of *hae hsl2* in *Arabidopsis* identified 277 genes to be significantly downregulated (≥2-fold, FDR < 0.05). These genes were enriched for the GO terms “carbohydrate metabolism”, “cell wall modification”, and “defense response”, including *PGAZAT/ADPG2* and *QRT2* (polygalacturonases), pectinesterases, XTHs, and cellulases that degrade the middle lamella as well as *FAR4*, *FAR5*, *GPAT5*, and phenylpropanoid biosynthetic enzymes responsible for the formation of protective suberin and lignin layers. The qPCR and cluster analysis show a peak expression at late stage 15, placing them downstream of IDA-HAE/HSL2. The absence of these downregulated genes explains the non-abscission phenotype of *hae hsl2* mutants in *Arabidopsis* (Niederhuth et al., 2013b).

#### 4.4.2 HSL1

ET is usually antagonistic or synergistic with IAA. The ET–IAA antagonism in litchi fruitlet leads to its abscission. In litchi, LcARF5 (Auxin Response Factor 5) could upregulate both *LcIDL1* and *LcHSL2*, and *LcEIL3* (*Entheleny Insensitive like 3*) could only upregulate *LcIDL1* (Ma et al., 2024). This discovery of ET and IAA directly interacting with the IDA-HAE/HSL2 module is awaiting further confirmation in other species and organs.

#### 4.4.3 HSL3

As discussed earlier, HSL3 and its ligands can be induced by ABA, thereby regulating stomatal closure and contributing to drought tolerance. By recognition of CTNIP peptides, HSL3 triggers MAPK cascade and ROS-dependent transcriptional reprogramming enriched in defense and hormone-responsive genes. The CTNIP-HSL3 pathway is inducible by MAMPs, ET, and ABA, marking the receptor’s role at the crossroads of hormone-mediated stress reactions (Liu et al., 2020; Rhodes et al., 2022).

### 4.5 Functional validation of HAESA family proteins in multiple plants

#### 4.5.1 Abscission of flowers, leaves, and fruits

The function of regulating the abscission process of HAESA family proteins is the most conservative function in different plants (Wang et al., 2023). We have already confirmed related functions in soybean (*Glycine max*), tobacoo (*Nicotiana benthamiana*), tomato (*Solanum lycopersicum*), citrus (*Citrus clementina*), litchi (*Litchi chinensis*), oil palm (*Elaeis guineensis*), rose (*Rosa bourboniana*), etc.

In citrus, homologs of IDA regulate floral organ abscission. The insight into the role of citrus IDA demonstrated that CitIDA3 shares both structural and functional similarity with AtIDA, supporting the notion of conserved structure and function of IDA across plant species (Estornell et al., 2015; Magalhaes et al., 2016; Guo et al., 2022). Similarly, an investigation between IDA family peptides and HAE family receptors in tobacco found that this signaling module regulates corolla abscission through a mechanism similar to that in *Arabidopsis* (Ventimilla et al., 2020, 2021). RbIDL1–4 in the rose cultivar *Rosa bourboniana* “*Gruss an Teplitz*” was identified, in which RbIDL1 and RbIDL4 exhibited expression patterns and functions similar to those of *Arabidopsis*, acting as key components under ethylene regulation during petal abscission in rose (Singh et al., 2023).

Despite the phenotype observed in floral organs, the IDA-HAE/HSL2 module might contribute to the shedding process of leaves and fruit (Li and Su, 2024). LcIDL1 and LcHSL2 are vital to fruitlet abscission in litchi, which are both upregulated by transcription factor LcARF5, while LcEIL3 could only upregulate the expression of LcIDL1 as mentioned (Wang et al., 2019a; Ma et al., 2024). Meanwhile, IDA peptides were reported to promote the abscission of leaf and fruit in both *Populus* and oil palm trees, further supporting IDA-HAE/HSL2 module’s conservation in different species of plants and AZs of different organs (Tranbarger et al., 2019).

However, the functions of the HAESA family proteins also exhibit species-specific characteristics—for example, the presence of an IDA-HAE signaling module was discovered specifically in the AZ of tomato flowers. Among the ligands, SlIDA and SlIDL2~5 are upregulated during natural abscission, whereas SlIDL6/7 shows a stronger induction under stress conditions, indicating a dual regulatory role in balancing developmental and stress-induced abscission (Lu et al., 2023).

#### 4.5.2 The dehiscence of silique

Rapeseed (*Brassica napus*) and soybean (*Glycine max*) are the main oil crops cultivated worldwide, which are crucial to the global food security (Geng et al., 2022). However, excessive silique or pod dehiscence can result in considerable yield losses, making it one of the most important agronomic traits targeted in breeding programs for oil crops (Liu et al., 2024; Zhou et al., 2025). Due to the IDA-HAE/HSL2 module mediating organ abscission and both HAE and HSL2 expressing in the dehiscence zone (DZ) of mature silique in *Arabidopsis* (Kumpf et al., 2013; Sto et al., 2015), it is possible that HAESA family proteins also participate in regulating silique or pod dehiscence in rapeseed or soybean. Further investigation of HAESA family proteins is likely to provide insights for developing cultivars for large-scale mechanical harvesting (Yu et al., 2020; Li et al., 2021b; Parker et al., 2021; Geng et al., 2022, 2025; Nichol et al., 2025).

## 5 Discussion

Among all of the functions of HAESA family proteins, IDA-HAE/HSL2 plays the most important role mainly in abscission process, providing a signaling module in which the abscission is triggered by peptides, recognized by receptors and co-receptors, amplified by the MAPK cascade, and finally controlled and performed by the genes inside the cell nucleus. Many other proteins also assist the transmission of this signal on different aspects, including interfering transcription factors and membrane vesicle transportation (Shan et al., 2021). Some other tools such as the scaffold protein BSK1 and the “signal grounding wires” MKP1 are equally important because they ensured the pathway to be activated in appropriate time and intensity (Galindo-Trigo et al., 2024; Taylor et al., 2024). Aside from abscission, the HAESA family proteins are also involved in plant development and stress responses (Ou et al., 2021). The HAESA family proteins are crucial to LRE and could regulate the renewal of root caps, which hinted their relations with drought resistance (Kumpf et al., 2013; Shi et al., 2018; Zhu et al., 2019; Zhang et al., 2022). The HAESA family proteins are also related to plant immunity by both regulating the separation of infected leaves and upregulating resistance genes, which indicates the dual strategy of plants when they are subjected to various stresses (Qian et al., 2018; Liu et al., 2022; Rhodes et al., 2022; Lalun et al., 2024).

It is widely accepted that HAESA family proteins have ligand diversity and specificity. In the IDA-HAE/HSL2 module, the high binding affinity between the ligand and the receptor might be the insurance of the correct activation of the IDA signal. However, those ligands which bind with HAESA family proteins with low affinities might bind with other receptors and *vice versa* (Roman et al., 2022)—for instance, HSL2 could recognize IDL1, and CLE9 could also be sensed by HSL1 by the assistance of SERK1 (Qian et al., 2018). The receptor recognition processes are diverse, which is possibly due to the structure similarity. There comes a new approach which might fit for receptor function inspection: screening small molecules that could competitively inhibit the ligands. This method is applied in FER by screening a bank of kinase inhibitors and making several crops acquire disease resistance (He et al., 2016; Zafar et al., 2019; Guo et al., 2020; Zhang et al., 2020b; Liu et al., 2023). In further research, bioinformatics methods such as virtual screening and molecular dynamic simulation could reduce the cost of high-throughput screening, probably contributing to the study on signal recognition and transmission.

Cooperation between elements in the abscission regulatory network still needs to be discovered. Various peptides or signals such as IDA/IDL, CLE, and CTNIP/SCREW induced by different tissues and stresses should be regularly allocated in appropriate time and space (Sarwar et al., 2023). Moreover, more parts of the signaling pathway should be quantified, including membrane transportation, expression quality control, and signal strength, such as determining how the ER and the Golgi jointly set the effective copy number and threshold for HAESA family proteins on plasma membrane.

The HAESA family proteins function not only as a “switch” for abscission but also as a central hub for integrating diverse environmental signals. A previous research reported that the IDA-HAE/HSL2 module could promote drought tolerance by inducing leaf abscission or modulating stomatal movement, thereby reducing the transpiration surface area and water loss (Qian et al., 2018; Li et al., 2022; Liu et al., 2022; Rhodes et al., 2022). However, the very same signaling pathway may trigger whole-leaf shedding or upregulate defense-related genes in response to pathogen invasion, serving as a strategy to restrict the spread of diseases. This dual-function highlights the dynamic trade-offs between “retaining the leaves for growth” and “sacrificing tissues for survival”. Drought response and immune response often display antagonistic interactions: for instance, excessive activation of immune pathways under water limitation can impair growth, while immune induction may interfere with stomatal closure and exacerbate water loss (Zhang et al., 2016). This shows that the HAESA family proteins might emerge as coordinators in balancing resource allocation. Future research should employ transcriptomics and P–P interaction analysis to reveal how these receptors establish signaling priorities under distinct environmental situations, thereby revealing how plants optimize the balance between growth and survival with limited resources.

In crop production, organ abscission exerts a double-edged effect: premature shedding may cause yield loss, whereas appropriate shedding could reduce post-harvest waste. Given that the IDA-HAE/HSL2 module is highly conserved in multiple species yet exhibits spatial and temporal specificity, its potential applications in crop improvement is huge—for example: in *Brassica napus*, the IDA-HAE/HSL2 module may be related to the cracking of the siliques (Xu et al., 2019; Li et al., 2021a; Qing et al., 2021). In fruit trees such as citrus and litchi, the same module is tightly related to the drop of flowers and fruitlets. By employing abscission zone-specific promoters to drive the expression of receptors or ligands, it is possible to achieve precise regulation, ensuring stable fruit retention before harvesting while enabling rapid abscission afterward (Zhao et al., 2023a; Xia et al., 2024). In addition, inducible promoters can be employed to restrict the temporal window of receptor activity, ensuring that signaling is activated only during harvest or under stress conditions. Overall, gene editing combined with molecular design holds great promise to achieve tissue- and stage-specific regulation, thereby enabling precise control without compromising yield or quality.

Further research could be focused on three aspects: (1) revealing more synergistic and antagonistic interactions among different participants in HAESA family network under multiple stress conditions, (2) quantifying the mechanisms including membrane transportation, protein folding checks, and signal intensity, and (3) by applying the methods of molecular breeding, maximizing the potential of the HAESA family proteins to balance the trade-off between growth and stress responses. In summary, the HAESA family proteins represent not only a pivotal subject in basic plant biology but also a critical leverage point for future crop improvement and sustainable agriculture.
